# Addressing depression in older adults with Alzheimer’s through cognitive behavioral therapy: systematic review and meta-analysis

**DOI:** 10.3389/fnagi.2023.1222197

**Published:** 2023-09-13

**Authors:** Ana María González-Martín, Agustín Aibar Almazán, Yulieth Rivas Campo, Noelia Rodríguez Sobrino, Yolanda Castellote Caballero

**Affiliations:** ^1^Department of Education and Psychology, Faculty of Social Sciences, University of Atlántico Medio, Las Palmas, Spain; ^2^Department of Psychology, Centro de Educación Superior de Enseñanza e Investigación Educativa, Madrid, Spain; ^3^Department of Health Sciences, Faculty of Health Sciences, University of Jaén, Jaén, Spain; ^4^Faculty of Human and Social Sciences, University of San Buenaventura-Cali, Cali, Colombia

**Keywords:** cognitive behavioral therapy, depression, Alzheimer’s, older adults, systematic review, meta-analysis

## Abstract

**Objectives:**

This systematic review and meta-analysis was conducted to provide an analysis of the published data about the effects of cognitive behavioral therapies on the depression of older adults with a diagnosis of Alzheimer’s disease.

**Methods:**

This study was performed following the PRISMA 2020 guidelines. The search was performed between March and April 2023, using four electronic databases: PubMed, Web of Science, Cinhal and Scopus. Different keywords combined with Boolean operators were utilized. Only 11 articles were included out of the initial 212.

**Results:**

Cognitive behavioral therapy was found to reduce depression in individuals with Alzheimer’s, including treatments with low frequency but a longer intervention time.

**Conclusion:**

This systematic review and meta-analysis found that the psychosocial therapy cognitive behavioral therapy is effective in improving depression in individuals with a diagnosis of Alzheimer’s. However, results are inconclusive due to the disparity of the findings and the heterogeneity of the applied protocols, so more studies are needed on this topic.

**Systematic review registration:**

https://www.crd.york.ac.uk/prospero/display_record.php?RecordID=416396, CRD42023416396.

## 1. Introduction

The number of people aged ≥65 is projected to increase by 1,250 million by 2050 (Buschert et al., 2010), with an estimated 115.4 million people living with dementia (Form the Brain Consortium, 2017). Alzheimer’s disease (AD) is the cause of 60–70% of dementia, affects 48 million people worldwide (Li and Liu, 2016) being a significantly higher number of people compared to other types of dementias. Since it affects a large number of people around the world, understanding and addressing this disease has a significant impact on public health and the quality of life of affected people and their caregivers (Prince et al., 2013).

AD, which is characterized by cognitive impairment, behavioral alterations and decreased activities, is a progressive neurodegenerative disease (Li et al., 2016). The prevalence of person living with dementia doubles approximately every 6 years from age 65, reaching 7% in people aged 75–79 years, 12% in people aged 80–84 years, 20% in people aged 85–89 years, and 40% in those older than 90 years (Alzheimer’s Disease International, 2015). AD can be identified as a global public health problem in the coming seasons, with more than 20 million people affected worldwide (Association As, 2013).

Person living with AD may experience cognitive and functional losses during the course of the disease as well as problems in both mental health and performing the activities of daily living (Cipriani et al., 2020; Jia et al., 2021). This loss of functionality can lead to greater dependency on caregivers and affect the autonomy and quality of life of people (Alcañiz et al., 2015). Within the psychological disorders derived from the disease, we find that depression is common among people affected by AD, both at the beginning of cognitive decline and later, when the dementia process is more severe (Tarawneh and Holtzman, 2012).

The prevalence of depression in AD is between 38 and 50% (Byers and Yaffe, 2011; Chi et al., 2015), meaning that approximately two and a half million individuals may have depression in various phases of AD. The prevalence of depression increases in mild–moderate phases of AD (Zhao et al., 2016). The presence of depression in people with this disease increases the risk of behavioral alteration and accelerates functional deterioration (Lyketsos et al., 1996). Furthermore, depression has been shown to be the most consistent risk factor associated with psychological or behavioral symptoms and cognitive decline in people with AD (The Consortium for the Early Identification of Alzheimer’s Disease – Quebec et al., 2020). Despite the importance of depressive symptoms in people with AD, there is little evidence of the efficacy of pharmacological interventions to try to counteract these symptoms, as permanent successful results have not yet been achieved (Salloway et al., 2014; Vandenberghe et al., 2016). Antidepressant medication has been discussed as an important approach to treating depression in AD (Correia and Vale, 2021), but not all individuals respond to drug therapy (Al-Harbi, 2012). Additionally, the management of depression in these people is complicated by comorbid medical conditions, potential drug interactions, increased vulnerability to the side effects of medications typically used to treat depression and medication costs (Lyketsos et al., 2002; Ionescu et al., 2015). Therefore, a psychosocial intervention aimed at treating depression in people with AD may be a reasonable alternative that is preferable to modifying the patterns of daily life that may cause and maintain reactive symptoms of depression (Buschert et al., 2010). Additionally, older adults generally tend to prefer talking therapies (Chilvers et al., 2001; Landreville et al., 2001).

Psychosocial interventions include Cognitive Behavioral Therapy (CBT), which is being used to improve depressive symptoms in people living with AD and is characterized by a therapeutic approach based on the idea that thoughts, emotions and behaviors are interrelated and that, By identifying and changing negative or dysfunctional thought patterns and behaviors, people’s emotional states and quality of life can be improved (Orgeta et al., 2015). In the context of AD, scientific evidence reports that CBT can be adapted to address emotional issues, such as anxiety and depression, and to help people adjust to the cognitive and functional challenges that the disease brings (Bird and Blair, 2010; Driessen and Hollon, 2010; Spector et al., 2012; García-Alberca, 2017).

Based on this, the purpose of this systematic review and meta-analysis is to provide an analysis of published data about the effects of cognitive behavioral therapies on the depression of older adults diagnosed living with AD.

## 2. Materials and methods

This systematic review and meta-analysis focused on exploring the effect of cognitive behavioral therapies (CBT) on the depression of older adults with a diagnosis of AD. It was carried out under the guidelines of the PRISMA 2020 document, using the protocol prespecified in the CRD42023416396 registry in PROSPERO. Additionally, the methodological recommendations from the Cochrane Manual for the Development of Systematic Reviews of Interventions proposed by Higgins et al. were utilized (Higgins et al., 2011).

### 2.1. Information sources and search strategy

Data collection was carried out in the months of March and April 2023, using the PubMed, Web of Science, Cinhal and Scopus databases. The Boolean operators “AND” and “OR” were used to connect keywords in the following search equation: (“Cognitive Behavioral Therapy” OR “Cognitive Behavior Therapies” OR “Cognitive Psychotherapy” OR “Cognitive Psychotherapies”) AND (“depression” OR “mental health” OR “Anxiety” OR “Distress”) AND (“Alzheimer Disease” OR “Alzheimer”).

### 2.2. Inclusion criteria

Included articles had to meet the following criteria: (i) Pacientes: Older individuals with a diagnosis of AD. No limitations were imposed based on age, gender, or race; (ii) different types of clinical trials included Cognitive behavioral therapy vs. placebo, TAU, usual standard clinical care, Baseline treatments; (iii) Primary outcome measures: depression. CSDD: The Cornell Scale for Depression in Dementia; GDS: Geriatric Depression Scale; BDI: Beck Depression Inventory; MADRS: Montgomery-Åsberg Depression Rating Scale; (iv) Published in English or Spanish.

### 2.3. Exclusion criteria

(i) Literature that has been repeatedly published by the same author or contains duplicate data; (ii) AD coexisting with other organic illnesses; (iii) the protocols, Pilot studies, registries, studies that did not measure relevant study variables or that focused on people of ethnic minority backgrounds were excluded.

### 2.4. Study selection process

The selection of articles was made using the virtual tool Rayyan^1^^,^^2^, through which the articles found in each database were consolidated and duplicates eliminated. The screening was performed by title and abstract, with those that met the established criteria classified as included. Two investigators oversaw the inclusion decision, ensuring blinding between them. In case of discrepancies, a third author decided to include or exclude the article.

### 2.5. Data extraction

We extracted year of publication, country and author, characteristics of the participants (age, sample size and distribution of the group), intervention to be followed by the experimental and control groups (duration of the intervention, duration of each session and frequency), type of variable, test used, follow-up time and the statistical value from each selected article.

### 2.6. Methodological quality assessment

The PEDro scale was used to assess the quality of the selected articles. This scale is composed of 11 items that evaluate the internal and external validity and statistical support of a publication (Cashin and McAuley, 2020). We evaluated items 2–11, awarding each a score of zero to one, according to whether it appears (one) or not (zero) within the publication, with the exception of the first one, which is related to external validity, so each publication could achieve a minimum score of zero and a maximum of ten. Those with a score of fewer than four points were rated as “poor,” four to five points as “average,” between six and eight points as “good” and between nine and ten points as “excellent” (de Morton, 2009). The same as recorded in the Study Selection Process, two researchers were responsible for the decision; in case of discrepancies, a third author defined.

### 2.7. Analytical decisions for meta-analysis

We performed a meta-analysis of data for the mean and standard deviation of changes over time of the observed variables by applying a random effects model. Where SD was not available, 95% CI was used. The results of the meta-analysis are shown in the form of a forest plot of included articles, showing the first author, the date of publication, the sample size, the individual effects (with Hedge’s *g*) and the overall effect with the 95% CI, as well as the value of *p* associated with the statistic. Studies were grouped according to the conditions of variability, while the effect size and variability within each were calculated, with analysis stratified by subgroup. To address potential publication bias, graphical analyses were performed using funnel plots and their distribution.

## 3. Results

### 3.1. Selection of studies

**Figure 1:**
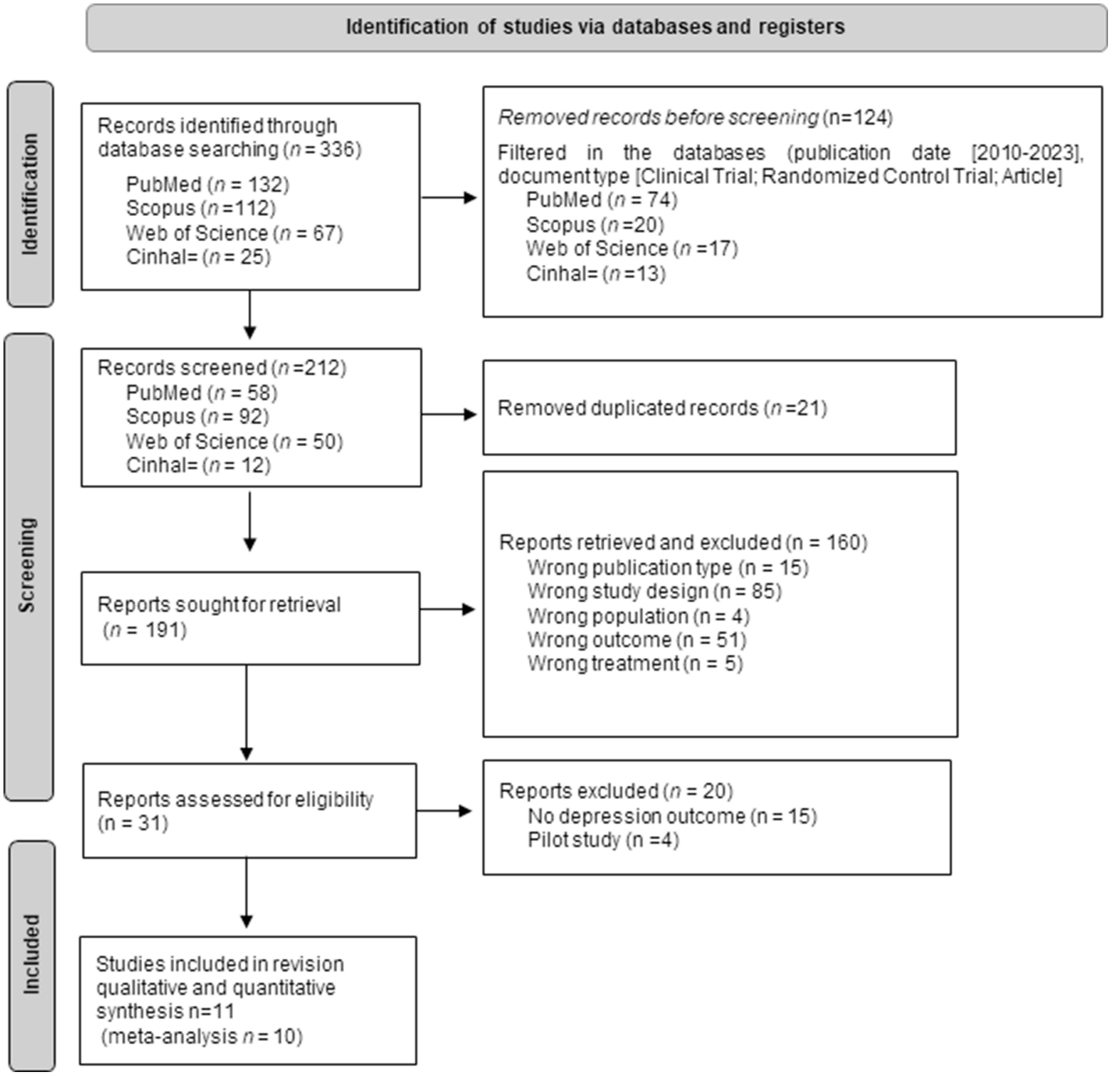
Flow chart identification of studies *via* databases and registers.

A complete search was carried out across the aforementioned databases, resulting in a total of 212 articles. Duplicate records were eliminated and a filter applied to select studies from the last 10 years. Only 11articles met the inclusion criteria (Bergamaschi et al., 2013; Gaitán et al., 2013; Amieva et al., 2016; García-Casal et al., 2017; Giovagnoli et al., 2017; Yang and Kwak, 2017; Lök et al., 2019; Kim, 2020; Tonga et al., 2021; Bartels et al., 2022; Justo-Henriques et al., 2023; Figure 1).

### 3.2. Methodological quality

Methodological quality was evaluated through the PEDro scale. Scores for two of the included articles (Kim, 2020; Tonga et al., 2021) were found on the PEDro website, while the other nine (Bergamaschi et al., 2013; Gaitán et al., 2013; Amieva et al., 2016; García-Casal et al., 2017; Giovagnoli et al., 2017; Yang and Kwak, 2017; Lök et al., 2019; Bartels et al., 2022; Justo-Henriques et al., 2023) were calculated manually. None of the included articles were of poor methodological quality. Five studies (Gaitán et al., 2013; Giovagnoli et al., 2017; Kim, 2020; Bartels et al., 2022; Justo-Henriques et al., 2023) were of average quality, while the remaining six were of good methodological quality (Bergamaschi et al., 2013; Amieva et al., 2016; García-Casal et al., 2017; Yang and Kwak, 2017; Lök et al., 2019; Tonga et al., 2021) (Table 1).

**Table 1:** Methodological quality of the articles included.

Item authorship	1	2	3	4	5	6	7	8	9	10	11	Total
Tonga et al. (2021)	Yes	Yes	Yes	Yes	No	No	Yes	No	Yes	Yes	Yes	7
Kim (2020)	Yes	Yes	No	Yes	No	No	No	Yes	No	Yes	Yes	5
Amieva et al. (2016)	Yes	Yes	Yes	No	No	Yes	No	Yes	No	Yes	Yes	6
Giovagnoli et al. (2017)	Yes	Yes	No	Yes	No	No	Yes	No	No	Yes	Yes	5
García-Casal et al. (2017)	Yes	No	No	Yes	Yes	No	No	Yes	Yes	Yes	Yes	6
Bartels et al. (2022)	Yes	No	No	Yes	No	No	No	Yes	No	Yes	Yes	4
Lök et al. (2019)	Yes	Yes	No	Yes	No	No	Yes	No	Yes	Yes	Yes	6
Justo-Henriques et al. (2023)	Yes	Yes	No	Yes	No	No	Yes	No	No	Yes	Yes	5
Yang and Kwak (2017)	Yes	Yes	Yes	No	Yes	No	No	No	Yes	Yes	Yes	6
Bergamaschi et al. (2013)	Yes	Yes	No	Yes	No	No	Yes	Yes	Yes	Yes	Yes	7
Gaitán et al. (2013)	Yes	Yes	No	No	No	No	Yes	No	No	Yes	Yes	4

Items: 1, eligibility criteria; 2, random allocation; 3, concealed allocation; 4, baseline comparability; 5, blind subjects; 6, blind therapists; 7, blind assessors; 8, adequate follow-up; 9, intention-to-treat analysis; 10, between-group comparisons; 11, point estimates and variability; Y, yes; N, no. The eligibility criteria item does not contribute to the total score.

### 3.3. Study characteristics

The articles included in this systematic review and meta-analysis were all randomized controlled clinical trials published in Korea (Yang and Kwak, 2017; Kim, 2020), Norway (Tonga et al., 2021), France (Amieva et al., 2016), Spain (Gaitán et al., 2013; García-Casal et al., 2017), Portugal (Justo-Henriques et al., 2023), Italy (Landreville et al., 2001; Giovagnoli et al., 2017), Turkey (Lök et al., 2019), and Germany (Bartels et al., 2022).

A total of 861 people aged between seventy and eight years participated in the included studies. The duration of the CBT interventions varied between 45 and 60 min, with programs between 2 and 24 months. Interventions were contrasted mainly with a traditional treatment control group. The main tools used to assess depression were the Geriatric Depression Scale (GDS), the Montgomery-Åsberg Depression Rating Scale (MADRS), the Cornell Scale for Depression in Dementia (CSDD) (Bergamaschi et al., 2013; Lök et al., 2019) and one study measured outcomes using the Beck Depression Inventory (Giovagnoli et al., 2017). GDS has been designed and validated primarily for use in geriatric populations in general, including older adults with and without AD. The GDS consists of a series of questions or statements related to depressive symptoms. The respondent is asked to answer “yes” or “no” to each statement, indicating whether they have experienced the described feelings or behaviors over the past week. The scale typically contains 15–30 items, depending on the version being used (Gaitán et al., 2013; García-Casal et al., 2017; Yang and Kwak, 2017; Kim, 2020; Bartels et al., 2022; Justo-Henriques et al., 2023). The (MADRS) consists of 10–15 items that assess various aspects of depression, such as sadness, apathy, inability to feel, fatigue, suicidal thoughts, and other symptoms associated with depression. The items are rated on a scale of 0 to 6 or 0 to 7, depending on the version used, where represents the absence of symptoms, and higher values indicate greater severity of depressive symptoms (Amieva et al., 2016; Tonga et al., 2021); The CSDD consists of 19 items that assess various aspects of depression, including mood-related symptoms, physical signs, behavioral disturbances, and cyclical functions. The scale is specifically adapted for use with individuals who have dementia and takes into account their communication and cognitive abilities (Bergamaschi et al., 2013; Lök et al., 2019). And the BDI consists of 21 multiple-choice items, each representing a specific symptom of depression. The individual is asked to rate how much each symptom has bothered them in the past 2 weeks. The total score obtained from the BDI indicates the level of depression experienced, with higher scores indicating more severe depressive symptoms (Giovagnoli et al., 2017).

### 3.4. Results of the study

Five studies (Yang and Kwak, 2017; Lök et al., 2019; Kim, 2020; Bartels et al., 2022; Justo-Henriques et al., 2023) found statistically significant favorable results for CBT intervention for depression in people with a diagnosis of AD. These findings were evident even in low-frequency treatments (only once a week) (Giovagnoli et al., 2017; Lök et al., 2019; Justo-Henriques et al., 2023), although these interventions took place over a sustained period, e.g., 8 weeks (Lök et al., 2019), 12 weeks (Giovagnoli et al., 2017), and 47 weeks (Justo-Henriques et al., 2023).

Depressive symptoms, as measured by the GDS, significantly decreased over time (*p* = 0.007, Cohen’s *d* = 0.39). Scores for the intervention group (IG) were significantly lowered to 0.55 ± 0.2, compared with 0.8 ± 3.9 for the basic test group (*p* = 0.028) (Yang and Kwak, 2017). Results in favor of the computer-based cognitive training (CBCT) group demonstrated changes in depression scores, with small effect sizes ranging from 0.21 to 0.36, but not statistically significant (*p* = 0.63) (Gaitán et al., 2013).

A group treatment of cognitive rehabilitation and cognitive-behavioral treatment for early dementia (CORDIAL) is feasible in a clinical routine setting and demonstrated antidepressant effects in the CBT IG compared with regular care (Bartels et al., 2022). A CBT program with musical and artistic stimuli achieved changes at the end of 24 sessions compared to initial values (*p* = 0.013) (Kim, 2020). Likewise, in 12 weeks, Giovagnoli et al. (2017) used the Beck Depression Inventory to verify that a lower level of depression can be reached for the IG compared to the control group (CG) (*p* = 0.015). This research highlights that, in participant with AD, CBT can improve initiative and stabilize memory, while non-cognitive treatments can improve psychosocial aspects.

However, among the investigations that used the MADRS scale for the evaluation of depression, there were no significant differences in the group x time interaction. Among these is the Norwegian study (Tonga et al., 2021), where the regression coefficient in the GI is −1.31 (0.83) and a non-significant interaction is reported (*p* = 0.34), and the French study (Amieva et al., 2016) (*p* = 0.916) (Table 2).

**Table 2:** Effects of CBT interventions in persons with a diagnosis of AD and depression.

Author and year	Country	Sample CG/IG	Control group	Intervention group					
				Age mean (SD)	Intervention type	Measuring instrument	Assessments	Depression results	Values post treatment
Tonga et al. (2021)	Norway	CG (*n* = 100) IG (*n* = 98)	TAU	70.1 ± 7.82	CORDIAL program comprising multimodal intervention: 11 individual sessions with CBT, cognitive rehabilitation and reminiscence therapy.	MADRS	T0 = Baseline T1 = over the course of 4 months T2 = 10-month follow-up visit	Depressive symptoms showed a significant improvement but no differences between-groups.	Difference in means 1.31 *p* = 0.08
Kim (2020)	Korea	CG (*n* = 17) IG (*n* = 18)	Regular activities at existing centers (physical activity, recreation, watching TV). Five times per week, 60 min per session, a total of 24 sessions.	IG = 80.6 (±5.1) CG = 77.88 (±5.49)	Recollection-based cognitive stimulus program (physical, horticultural, musical, art and IADL activity). Five times per week, 60 min per session, a total of 24 sessions.	GDS - Korean version	T0 = Baseline T1 = finalized and 24 sessions	Depressive symptoms in the experimental group significantly decreased	Difference in means 2.4 *p* = 0.031
Amieva et al. (2016)	France	CG (*n* = 153) IG (*n* = 156)	Usual medical care excluding non-drug therapy.	CG = 78.7 (6.5) IG = 78.9 (6.2)	Individualized cognitive rehabilitation therapy consisted of a made-to-measure program conducted through individual sessions. The first two sessions were exclusively dedicated to selecting meaningful activities. The activities to be trained had to be selected according to personally relevant goals for the participants.	MADRS	T0 = Baseline T1 = 3 months T2 = 24 months	No significant differences were found with post-measures.	Difference in means 0.58 *p* = 0.911
Giovagnoli et al. (2017)	Italy	CG (*n* = 13) IG (*n* = 13)	A non-verbal approach with free sound-music interactions, using rhythmical and melodic instruments, was adopted.	IG = 71.69 (7.88)	CT for 12 weeks, including two 45-min group sessions a week.	BDI	T0 = Baseline T1 = Follow-up at the end of treatment T2 = Follow-up 3 months after the end of treatment	Depressive symptoms showed a significant improvement and revealed a between-group difference	Difference in means 0.72 *p* = 0.002
García-Casal et al. (2017)	Spain	CG (*n* = 10)/CG TAU (*n* = 11) IG (*n* = 11)	TAU	IG = 76.73 (±5.57) CG = 75.80 (±6.48)	20 sessions of rehabilitation of emotion recognition and 20 sessions of CST.	GDS	T0 = Baseline T1 = 6 months	No significant differences were found with post-measures.	Difference in means −1,03 *p* = 0.62
Bartels et al. (2022)	Germany	*n* = 51	Baseline treatments.	72.4	10 bi-weekly sessions of CBT.	GDS	T0 = Baseline T1 = 6 months	Depressive symptoms as measured by the GDS significantly decreased over time	Difference in means 0.5 p = 0,007
Lök et al. (2019)	Turkey	CG (*n* = 30) IG (*n* = 30)	No treatment.	--	Therapy was applied once a week and lasted for 8 weeks. Every session took 60 min.	CSDD	T0 = Baseline T1 = 8 weeks	No significant differences were found with post-measures between groups	Difference in means 0.86 *p* = 0.45
Justo-Henriques et al. (2023)	Portugal	*n* = 59	Baseline treatments.	IG = 78.53 CG = 79.21	45 min, once a week, 47 weeks, 35 h total of ICS.	GDS	T0 = Baseline T1 = 25 weeks T2 = 50 weeks	The results showed a significant effect of the intervention	Difference in means 1.37 *p* = 0.000
Yang and Kwak (2017)	Korea	CG (*n* = 10) IG (*n* = 10)	No treatment.	71.1 (6.9)	60 min sessions for a total of 12 weeks of computer-based cognitive program.	GDS	T0 = Baseline T1 = 12–14 weeks	The results showed a significant effect of the intervention	Difference in means 0.55
Bergamaschi et al. (2013)	Italy	CG (*n* = 16) IG (*n* = 16)	Pharmacological treatment and repeated cycles of non-specific cognitive exercises.	IG = 78.19 (5.50) CG = 77.72 (5.06)	Pharmacological treatment and repeated cycles of CT for 1 year. The experimental (i.e., combined treatment) group participated in 5 1-month cycles of CT (1 cycle: 20 sessions, 2 h per day, 5 days a week) with a break of 4 weeks in between each cycle.	CSDD	T0 = baseline T1 = 1 year at the end of the study	No significant differences were found with post-measures.	Difference in means 1.8 *p* = 0.05
Gaitán et al. (2013)	Spain	CG (*n* = 16) IG (*n* = 23)	TCT.	CG = 76.00 (6.61) IG = 74.87 (4.89)	CBCT and TCT (CBCT + TCT) for 3 months	GDS	T0 = baseline T1 = 3-month follow-up T2 = 12 month follow-up	No significant differences	Difference in means 0.23 *p* = 0.63

IG, intervention group; CG, control group; T, type; D, duration; F, frequency; SD, standard deviation; CBCT, computer-based cognitive training; TCT, traditional cognitive training; CT, cognitive training; CSDD, The Cornell Scale for Depression in Dementia; GDS, geriatric depression scale; CDR, clinical dementia rating; IADL, instrumental activity of daily living; ICS, individual cognitive stimulation; MMSE, Mini-mental state examination; MoCA, Montreal cognitive assessment; TAU, treatment as usual group; CST, cognitive stimulation therapy; BDI, Beck Depression Inventory; NE, neuroeducation; MADRS, Montgomery-Åsberg Depression Rating Scale; CBT, cognitive behavioral therapy.

**Figure 2:**
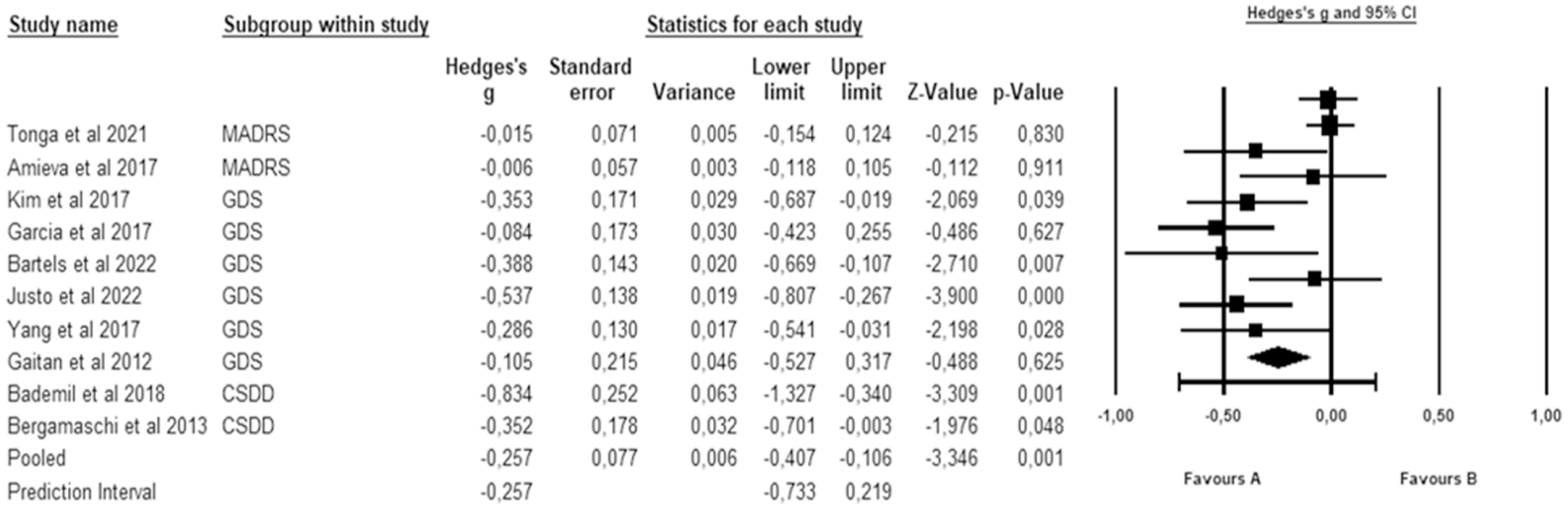
Effects of CTB over depression in people with a diagnosis of Alzheimer. The black box represents the point estimate for the respective study, while the size of the box represents the population size and the horizontal line is the 95% CI. The diamond-shaped figure represents the estimated point of the mean effect size.

Heterogeneity was tested by calculating the *I*^2^ and chi-square statistics. Given that the *I*^2^ value >50% and *p* < 0.1, which shows heterogeneity between the studies, a random effects model was used for the meta-analysis. The *I*-squared statistic is 72%, which tells us that some 72% of the variance in observed effects reflects variance in true effects rather than a sampling error. The forest plot shows the individual effects (with Hedge’s *g*) and the overall effect with the 95% CI, as well as the value of *p* associated with the statistic (Figure 2).

Through the meta-analysis, a significant but small effect size was observed of *g* = −0.257 (95% CI: −0.407 to −0.106; *p* = 0.001). If we assume that the true effects are normally distributed, we can estimate that the prediction interval is −0.733 to 0.219. The true effect size in 95% of all comparable populations falls in this interval.

**Figure 3:**
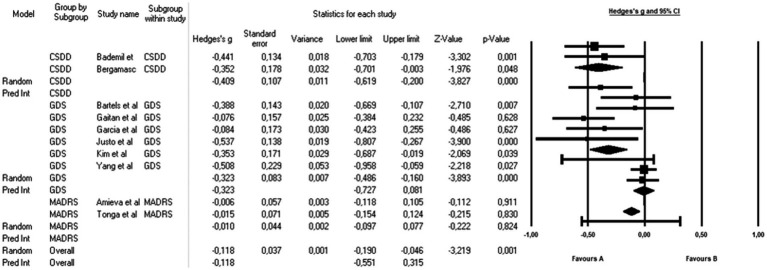
Subgroup analysis by measurement tools: effects of CTB over depression in people with a diagnosis of Alzheimer. The black box represents the point estimate for the respective study, while the size of the box represents the population size and the horizontal line is the 95% CI. The diamond-shaped figure represents the estimated point of the mean effect size.

To address the heterogeneity in the results, a subgroup analysis was performed which considered the differences in the depression measurement scales (Figure 3). The isolated analysis found that the influence of the CBT is evident in the decrease in depression evaluated with the CSDD with a small but significant mean effect size of *g* = −0.409 (95% CI: −0.619 to −0.2; *p* < 0.001); this same significant effect was found in studies in which depression was measured with the GDS *g* = −0.323 (95% CI: −0.486 to −0.160; *p* < 0.001).

### 3.5. Publication bias analysis

**Graph 1:**
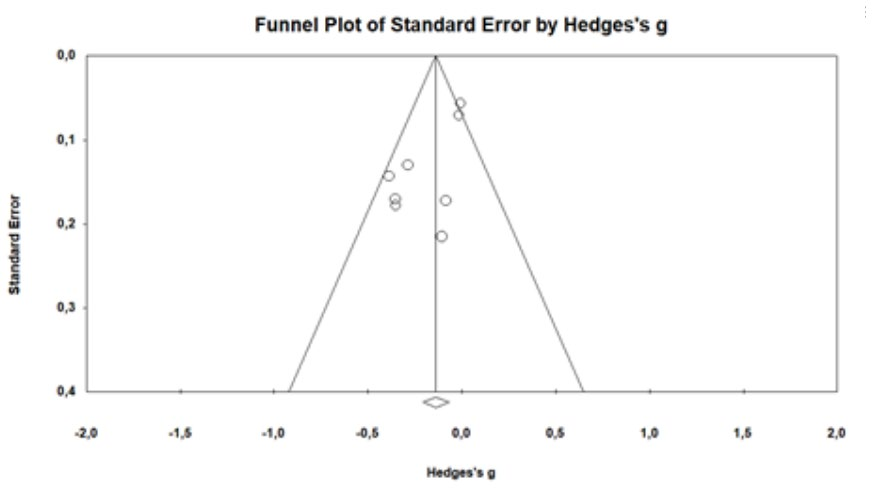
Funnel plot of standard error by Hedges’s *g*.

The analysis of the risk of publication bias was evaluated with a funnel plot that incorporated all the articles included in the meta-analysis. This revealed an expected publication bias given that some studies altered the size of the effect due to their differences in the significance of the results. However, when the analysis was performed excluding those studies a symmetrical distribution was maintained (Graph 1).

## 4. Discussion

The aim of this systematic review and meta-analysis was to determine the effects of cognitive behavioral therapy in older people with a diagnosis of AD. A total of 11 articles that met the inclusion criteria were included, where CBT was the main treatment and variables other than depression were evaluated to study the possible consequences that may aggravate the symptoms and progression of the disease (Bergamaschi et al., 2013; Gaitán et al., 2013; Amieva et al., 2016; García-Casal et al., 2017; Giovagnoli et al., 2017; Yang and Kwak, 2017; Lök et al., 2019; Kim, 2020; Tonga et al., 2021; Bartels et al., 2022; Justo-Henriques et al., 2023). The results of this systematic review suggest that CBT may be an effective intervention to reduce depression in people with a diagnosis of AD.

Cognitive behavioral therapy is a type of intervention that has attracted the attention of multiple researchers in recent years. Various studies have highlighted the effects of this intervention in participants with insomnia (Zhang et al., 2023), prostate cancer (Kemerer et al., 2023), type II diabetes mellitus (Abbas et al., 2023) and atherosclerotic cardiovascular disease (Li et al., 2023) among others. In addition, CBT has been observed to improve other psychological variables in participants with a diagnosis of AD, such as anxiety, a common coexisting complaint that has a significant negative impact on such participants (Kraus et al., 2008).

It is essential to highlight that no article included in this review was of excellent quality. Instead, the articles ranged in quality from average to good. One major problem was that most articles did not carry out an adequate allocation and only two studies (García-Casal et al., 2017; Yang and Kwak, 2017) blinded their participants, which could have altered the results. Inadequate concealment and lack of blinding of study participants have been associated with an exaggeration of results by 7 and 13%, respectively (Savović et al., 2012).

In general, the participants described in these studies had clinically valuable reductions in depression after participating in treatment, and in some studies, those reductions were maintained at follow-up (Gaitán et al., 2013; Tonga et al., 2021; Bartels et al., 2022). Additionally, the high adherence rate in the participation of these studies indicates that the treatment and evaluation protocol is feasible in this population. The mean number of sessions completed is remarkable given the frequent physical illnesses suffered by this population; the mean duration of each session indicated that the participants were able to maintain attention and participation in the treatment. This may be because CBT can be tailored to accommodate the limitations in understanding, learning, attention and self-control that are common in AD participants (Kraus et al., 2008). These positive findings were consistent with previous findings from other psychosocial interventions that significantly reduced depressive symptoms in people with a diagnosis of AD, such as mindfulness (Larouche et al., 2015; Paller et al., 2015; Quintana-Hernández et al., 2016; Marchant et al., 2021). Participants in these studies showed a significant improvement after the intervention, since the mean scores improved from the initial session to the following one. For example, Marchant et al. (2021) demonstrated that mindfulness-based treatment for participants with dementia of the Alzheimer type significantly reduced depression, an effect which was maintained at 6-month follow-up. In contrast, Quintana Hernández et al. (2015) showed that pharmacological treatment combined with mindfulness presented a better clinical evolution than pharmacological treatment alone or combined with relaxation. However, a systematic review (Olazarán et al., 2010) showed that most participants included in the studies do not qualify for clinical depression. Rather, they exhibited depressive symptoms, which indicates that more research is needed to reach a conclusion.

Importantly, three of the studies indicated in this systematic review and meta-analysis reported no statistically significant differences after the intervention (Bergamaschi et al., 2013; Amieva et al., 2016; Lök et al., 2019). However, this discrepancy could be explained by the use of different measures of depression outcome measures: studies by García-Casal et al. (2017), Kim (2020), and Bartels et al. (2022) used the GDS, which only included yes/no questions. In contrast, Amieva et al. (2016) used the MADRS, which consists of 10 items, each rated from zero to six based on clinical judgment of severity. Another explanation could be the disparity of the intervention programs, possible differences in the psychosocial interventions used or the baseline characteristics of the participants.

Additionally, different treatment modalities based on CBT with a diagnosis of AD continue to be studied. Within this systematic review, some studies use different application modalities. Gaitán et al. (2013) and Yang and Kwak (2017) found that the cognitive training programs using computers improved cognitive functions in several areas. Indeed, these authors hope that computer-based cognitive treatment will be more actively used in cognitive rehabilitation as it has several advantages over conventional group treatment. For example, the participant’s self-training and self-learning can shorten the therapist’s intervention time, and results are fed back to the participant immediately, motivating them to continue the treatment and obtain objective and precise results. Therefore, multimodal interventions that include the adoption of an active lifestyle should be recommended for older populations. Promoting lifestyle changes in the presymptomatic and predementia stages may have the potential to delay one-third of dementias worldwide (de la Rosa et al., 2020).

It should be noted that physical exercise plays an important factor in AD, although the results of existing scientific studies have not been consistent (Sobol et al., 2016). For example, in one study (van der Kleij et al., 2018), exercise did not lead to any improvement in cerebral blood flow, Aβ concentrations, total tau or phosphorylated tau concentrations in the cerebrospinal fluid of people with a diagnosis of AD (Steen Jensen et al., 2016). This indicates that although functional parameters are still likely to be positively affected in people with a diagnosis of AD, changes in brain function may require adjustment in different load components, such as duration of exercise sessions and frequency, intensity and type of exercise during the intervention. However, there is also evidence for the benefits of physical exercise. A meta-analysis that analyzed 122 studies synthesized the scientific evidence on the efficacy and safety of physical exercise as a complementary therapeutic intervention for quality of life, depressive symptoms and cognition (Dauwan et al., 2021). Therefore, future research should focus on both physical and cognitive treatments, seeking homogeneity in their application and even changing both treatments to see their effects on people with a diagnosis of AD.

This review also has some limitations. The main limitation was the small sample size, which was due to the study’s complexity and the limited availability of eligible participants for the present investigation, making it difficult to draw specific conclusions. Future prospective research with larger numbers of participants over longer follow-up periods is needed to confirm these promising findings and clarify the amount and types of activities that are most beneficial for participants with a diagnosis of AD and depression and to help maintain or improve their affective state and cognitive well-being. Another important limitation is the heterogeneity between the CBT interventions applied in the different studies, since they include variations in the content of the sessions, the modality of delivery (individual or group) and the total duration of the treatment. Also, differences in the control conditions used in the studies because some studies compared CBT with active treatments, others with treatment as usual, and others with control conditions involving some form of clinical treatment. These differences may have introduced bias and made it difficult to directly compare the effects of CBT in different settings. Other limitations were the mixed gender of the sample and the possible effects of comorbidities.

## 5. Conclusion

Depression affects a large proportion of AD people and has serious adverse consequences for both people and their caregivers. The present systematic review with meta-analysis revealed that CBT-based treatments appear to positively affect depression in people with a diagnosis of AD, although the results are inconclusive. This disparity in findings, together with the heterogeneity in the intervention protocols used, indicates that caution is needed before drawing any firm conclusions. Additionally, the available evidence regarding this therapy remains limited and the methodological quality of the evidence needs to be more rigorous. It is important to emphasize that more structured quality randomized controlled trials are still needed, with standardized and comparable protocols, a higher level of methodological quality and an adequate sample size to achieve a correct understanding of the effects of CBT on the short and long term in people with a diagnosis of AD. Likewise, more studies are required to determine if CBT is a better, worse or equivalent strategy to other types of psychosocial training.

## Data availability statement

The datasets presented in this study can be found in online repositories. The names of the repository/repositories and accession number(s) can be found in the article/supplementary material.

## Author contributions

AG-M and NR: conceptualization. AA and YR: methodology. AA and YC: writing – original draft preparation. YR and AG-M: writing – review and editing. NR and YC: supervision. All authors have read and agreed to the published version of the manuscript.

## Conflict of interest

The authors declare that the research was conducted in the absence of any commercial or financial relationships that could be construed as a potential conflict of interest.

## Publisher’s note

All claims expressed in this article are solely those of the authors and do not necessarily represent those of their affiliated organizations, or those of the publisher, the editors and the reviewers. Any product that may be evaluated in this article, or claim that may be made by its manufacturer, is not guaranteed or endorsed by the publisher.

## Footnotes

^1^ https://systematicreviewsjournal.biomedcentral.com/articles/10.1186/s13643-016-0384-4

^2^ https://rayyan.qcri.org/welcome
